# Outcome Measures Used in Ocular Gene Therapy Trials: A Scoping Review of Current Practice

**DOI:** 10.3389/fphar.2019.01076

**Published:** 2019-09-18

**Authors:** Jasleen K. Jolly, Holly Bridge, Robert E. MacLaren

**Affiliations:** ^1^Nuffield Laboratory of Ophthalmology, Nuffield Department of Clinical Neurosciences, University of Oxford, Oxford, United Kingdom; ^2^Wellcome Integrative Neuroimaging Centre, Nuffield Department of Clinical Neurosciences, University of Oxford, Oxford, United Kingdom; ^3^Oxford Eye Hospital, Oxford University Hospitals NHS Foundation Trust, Oxford, United Kingdom

**Keywords:** clinical trial, gene therapy, genetic eye disease, outcome measure, retinal imaging, vision, visual function

## Abstract

Multiple gene therapy trials are occurring for a variety of ophthalmic diseases around the world. The safety of gene therapy in the eye has been established, and the next step is to reliably assess efficacy. This is primarily done through the use of imaging techniques and visual function measures. Standardized visual function assessments, however, were originally developed for a clinical setting and may not be suitable for detecting and quantifying therapeutic changes. This scoping review takes a comprehensive look at current practice in terms of the outcome measures defined at trial registration. These were compared to the outcome measures reported in the literature. All published trials reported the pre-registered primary outcome measure. A range of additional secondary outcomes were reported that were not originally planned. Gaps in gene therapy assessment exist and further discussion are required to find a way forward, particularly as more conditions progress to phase 2 and 3 trials. Several factors impacting on trial design and outcome measure choice are discussed.

## Introduction

The eye presents the perfect organ for gene therapy. It is an immune privileged site, which is protected by the blood retinal barrier. The target cells, such as photoreceptors and retinal pigment epithelium are frequently non-dividing, meaning any intervention is likely to last for life. The different structures in the eye can be visualized due to the optical clarity inherent in the eyeball or can be imaged with well-documented techniques (Zysk et al., 2007; Fleckenstein et al., 2014). The structures in the eye can be targeted by various surgical procedures. Finally, many disease processes have a degree of symmetry. This means that, when treating one eye, the other eye can act as a control for comparison. For these reasons, ocular gene therapy is being trialed as an experimental treatment for an increasing number of conditions. There are established techniques to measure both structural and functional changes, with work ongoing in this field to evaluate the different diseases being treated. The success of gene therapy can be determined by the pattern of change seen in visual function measurement. Since visual function is the major marker for success of gene therapy, it is critical to establish guidelines for best practice.

Gene therapy can follow several different strategies. Most commonly, it is the supplementation of a defective gene with a working copy in affected target cells, as happens in achromatopsia and choroideremia. However, in some cases, such as with neovascular age-related macular degeneration, the gene expression may introduce a factor to help dampen the disease response. The mode of action is less important than the disease being investigated in determining the appropriate measures to use for trial monitoring. The Monaciano Symposium identified the measurement of treatment outcome as an area requiring priority review in order to aid the robustness of clinical interventional trials (Thompson et al., 2015). It calls for the investigation into appropriate outcome measures for each disease to measure structure and function without adding an unreasonable burden on the patient. They propose a standardization of testing protocols and data analysis. The reproducibility and reliability of tests should also be pre-defined.

With the increase in the number of trials using ocular gene therapy, the importance of adequate outcome measures is gathering interest. The success of gene therapy relies on three key aspects. The viral vector is developed over several years and optimized in animal models before reaching human trials (Koilkonda et al., 2014; Patrício et al., 2017). Much work has been conducted on optimizing the delivery of the therapeutic vector (Salvetti et al., 2017; Xue et al., 2017). Another component required for success of clinical trials is the adequate measurement of therapeutic impact. This requires a combination of the evaluation of ocular structure *via* imaging, and measurement of visual function. Standardized clinical visual function measures were largely developed for use in a clinical setting rather than for the assessment of novel interventions and may not always be adequate for measurement of a therapeutic effect. For example, the 100-hue test for color vision has wide normative ranges, making interpretation of longitudinal data difficult (Kinnear and Sahraie, 2002). The relationship of the outcome measures to disease progression, and therefore, the therapeutic window should also be better understood to interpret clinical trial findings. In addition, disease features such as visual field loss may make the conduct the test difficult.

A systematic review of gene therapy for retinal disease has been registered on the PROSPERO database (CRD42017056500) by London City University, but not yet completed. This specifies visual outcome as the outcome measure for assessing the success of trials, but the type of vision measure being looked at is not detailed, demonstrating the importance of providing further guidance on this topic. Additionally, as bilateral gene therapy will become more common, there will no longer be a control eye to provide a comparison as is done in many phase 1 trials, making vision outcome even more critical (MacLaren, 2016). Visual function is a combination of many aspects of vision, including detail, color, contrast, speed of vision, and night vision. The objective of this paper is to review the outcome measures listed and published for registered gene therapy trials in order to establish current practice, and to consider the scope for development of relevant outcome measures.

## Method

All clinical interventional trials must be registered on a publically available database. The databases on Clinicaltrials.gov (RRID: SCR_002309), EU clinical trials register (RRID SCR_005956), and the NIH clinical trials register were searched for all registrations by the end of October 2018, using the following search terms: gene therapy, subretinal injection, intravitreal injection, STX eye trial, Nightstar, Applied genetic, MeiraGTx, Hemera, Oxford Biomedica, Sanofi, Spark, ProQR, GenSight, and Genzyme.

Duplicate records were omitted from analysis. Natural history studies or studies specifically for long-term follow up of patients in a previous trial were also excluded in order to focus on the primary interventional trials. We then searched for any results from studies with a registered start date of greater than 12 months before October 2018. This was done *via* PubMed, study group websites, and Scopus. Searches were conducted using the investigator details and registered study name. Publications for the same study were grouped together and analyzed as an integrated dataset, with discrepancies between the primary outcome measure on the clinical trials record *versus* the final publications being noted.

## Results

### Listed Outcome Measures

We identified 50 unique clinical trials on the registers for 17 ophthalmic indications (**Supplementary Figure**). Lebers congential amaurosis, Leber hereditory optic neuritis, and choroideremia are the only conditions currently in phase 3 trials. Outcome measures were analyzed according to clinical trial phase and were separated into four categories: safety, validated tests, novel test methods, and non-specific (**Supplementary Table**).

Visual acuity was included in almost all studies as either a primary or a secondary measure. Various forms of perimetry also featured highly in the outcome measures list. Out of the 50 trials, 16 used broad descriptors which did not make clear what data were being collected or how it was going to be used. This included descriptors such as visual function or specifying imaging techniques with no details of the aspect of the images to be examined. One did not specify any outcome measures.

### Published Trials

One trial marked as completed has not yet been published (NCT00001735). Eighteen trials have associated results in the peer-reviewed literature. **Table 1** details the correlation between the trial register record and the outcomes reported in the peer-reviewed papers. Where primary and secondary outcomes were explicitly stated in the paper, these were recorded if also reported in the results. Anything not reported in the results or supplemental sections was not counted. In reports where primary or secondary was not made clear, all measures reported were recorded, and primary *versus* secondary was inferred from emphasis and context.

**Table 1:** Comparison of clinical trial record and published outcomes in ocular gene therapy trials.

Study ID (clinicaltrials.gov), disease and gene therapy delivery method	Phase; full/prelim	Pre-specified primary outcome	Reported primary outcome	Pre-specified secondary outcome	Reported secondary outcome
NCT01024998 Neovascular AMD AAV intravitreal (Heier et al., 2017)	I; full	Adverse events, maximum tolerated dose	Adverse events, change in VA and vector DNA concentration in biological samples	Decreased retinal thickness	Transgene expression in aqueous fluid and OCT thickness
NCT01494805 Neovascular AMD AAV subretinal (Rakoczy et al., 2015; Constable et al., 2017)	I/II; both	Adverse events and laboratory measures	Adverse events and laboratory measures	VA, foveal thickness, and CNV lesion	VA, retinal thickness, and standard injection retreatments
NCT01301443 Neovascular AMD lentivirus subretinal (Campochiaro et al., 2017)	I; full	Adverse events	Adverse events, change in VA, ocular inflammation, IOP, laboratory measures	OCT intraretinal fluid	Transgene expression, OCT macular thickening, lesion measures on fluorescein angiography, VA
NCT01461213 Choroideremia AAV subretinal (MacLaren et al., 2014; Edwards et al., 2016)	I/II; prelim	VA	VA	Microperimetry, OCT, and AF	Microperimetry threshold, OCT thickness, AF area
NCT02553135 Choroideremia AAV subretinal (Lam et al., 2019)	II; full	Adverse events	VA, adverse events	Macular autofluorescence, microperimetry	Microperimetry, contrast sensitivity, color vision, autofluorescence area, OCT ellipsoid zone and choroidal thickness assessments, safety
NCT02671539 Choroideremia AAV subretinal (Fischer et al., 2018)	II; full	VA	VA	Adverse events, autofluorescence, microperimetry, contrast sensitivity, color vision	Microperimetry, autofluorescence area, OCT ellipsoid zone and choroidal thickness, safety
NCT02077361 Choroideremia AAV subretinal (Dimopoulos et al., 2018)	I/II; full	Adverse events	Safety including adverse events	Microperimetry, Goldmann visual field, multifocal ERG, FST, OCT, photos, and autofluorescence	VA, autofluorescence area, OCT ellipsoid zone, microperimetry, quality of life questionnaire
NCT01482195 MERTK AAV subretinal (Ghazi et al., 2016)	I; full	Adverse events and laboratory measures	Safety measures	VA and FST	VA, FST, OCT thickness
NCT01267422 LHON AAV intravitreal (Wan et al., 2016)	Not given; prelim	VA, laboratory measures	VA, laboratory measures	IOP, neutralizing antibody assay, OCT RNFL thickness, computerized visual field mean deviation and visual field index, VEP, ERG, liver, and kidney function	Visual field index and mean deviation, VEP, OCT RNFL thickness, and blood tests
NCT02161380 LHON AAV intravitreal (Feuer et al., 2016)	I; prelim	Toxicity	Loss of VA	None	OCT RNFL thickness, pattern ERG, and adverse events
NCT01496040 LCA AAV subretinal (Le Meur et al., 2018)	I/II; full	Biodistribution in urine and nasal samples	Adverse events and biodsitribution	ERG, questionnaire, distance VA, near VA, color vision, pupillometry, microperimetry, and dark adaptation	Chorioretinal imaging, OCT thickness, undefined questionnaire, distance VA, nystagmus measures, visual field, microperimetry, fMRI, ERG, pupillometry, and mobility test
NCT00749957 LCA AAV subretinal (Weleber et al., 2016)	I/II; full	Adverse events	Adverse events	Static perimetry and VA	VA, static perimetry hill of vision, kinetic perimetry hill of vision, ERG, OCT, photography, and quality of life questionnaire
NCT00481546 LCA AAV subretinal (Hauswirth et al., 2008; A. V. Cideciyan et al., 2008; Artur V. Cideciyan et al., 2009; Jacobson et al., 2012; Artur V. Cideciyan et al., 2015)	I; both	Toxicity, symptoms, and, adverse events	Laboratory measures, symptoms, and adverse events	Visual function	VA, FST, dark adaptation kinetics, chromatic stimuli sensitivity, kinetic perimetry, OCT thickness, fixation analysis, pupillary light reflex, mobility testing, eye movements, and fMRI
NCT00516477 LCA AAV subretinal (Maguire et al., 2008, Maguire et al., 2009; Ashtari et al., 2011; Testa et al., 2013)	I; both	Safety and tolerability	Adverse events	Change in visual function psychophysical and objective measures	Pupillary light reflex, nystagmus testing, kinetic perimetry, microperimetry, OCT, AF, FST, ERG, mobility testing, and fMRI
NCT01208389 LCA 2^nd^ eyes (Bennett et al., 2016)	I/II; prelim	Adverse events	Adverse events	VA, VF, pupillary light response, mobility testing, FST, and contrast sensitivity	FST, kinetic perimetry, VA, pupillary light reflex, mobility, and fMRI
NCT00643747 LCA AAV subretinal (J W B Bainbridge et al., 2008; James W.B. Bainbridge et al., 2015; Ripamonti et al., 2015)	I/II; both	Inflammation	Adverse events	Visual function	Laboratory measures, VA, kinetic perimetry, microperimetry, dark-adapted perimetry, mobility, contrast sensitivity, color vision, spectral sensitivity, retinal imaging, and ERG
NCT00999609 LCA AAV subretinal (Russell et al., 2017)	3; full	Multi-luminance mobility testing bilateral	Multi-luminance mobility testing bilateral	FST, multi-luminance mobility testing monocular, and VA	FST, multi-luminance mobility testing monocular, VA, kinetic perimetry, Humphrey static visual fields, contrast sensitivity, and pupil light reflex
NCT02317887 XLRS AAV intravitreal (Cukras et al., 2018)	1; full	Adverse events, retinal structure, ocular structure	Adverse events, inflammation	Visual function, OCT, ERG, AAV antibodies	VA, microperimetry, ERG, OCT macular thickness, AAV antibodies

References provided in brackets. AF, fundus autofluorescence; AMD, age-related macular degeneration; ERG, electroretinogram; FST, full-field stimulus threshold; IOP, intraocular pressure; OCT, ocular coherence tomography; RNFL, retinal nerve fiber layer; VA, visual acuity; VEP, visual evoked potential.

**Figure 1:**
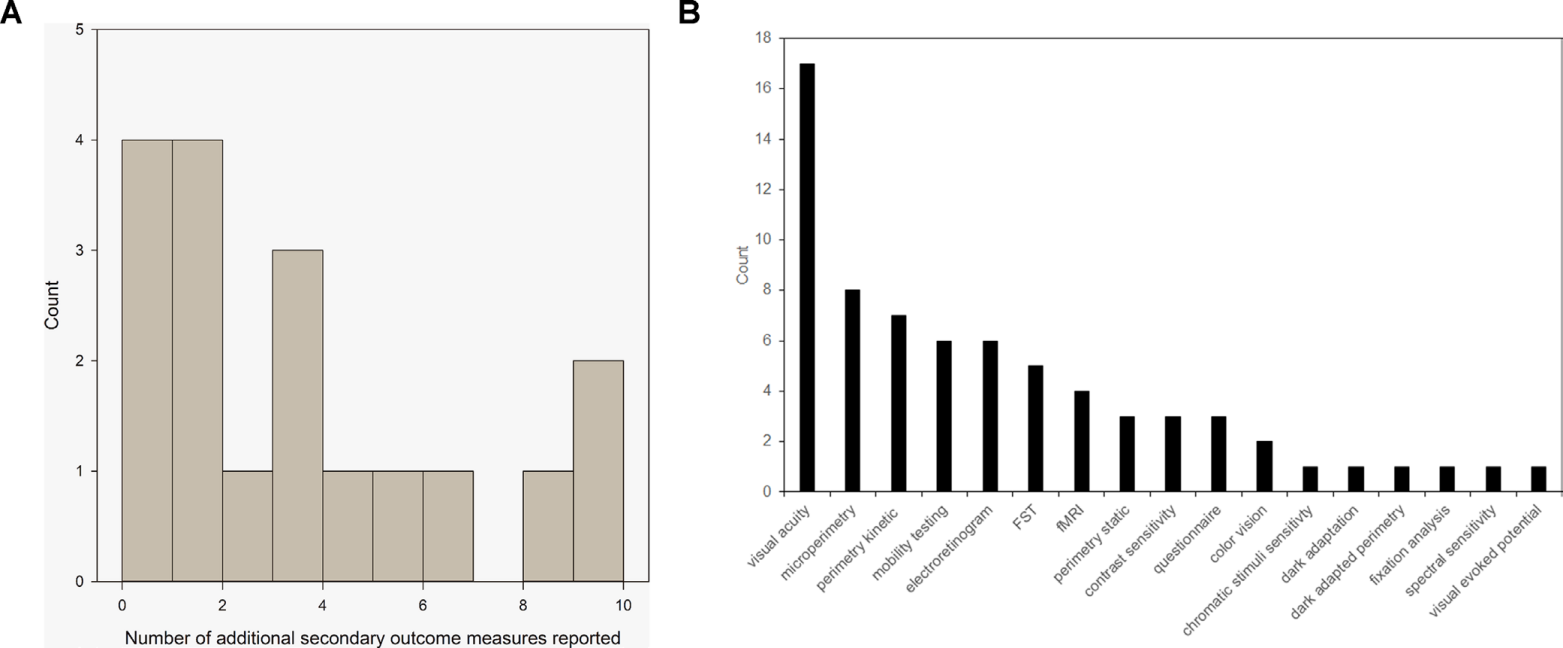
**(A)** Histogram to show the number of additional outcome measures reported by 79% of trials with peer-reviewed publications. **(B)** Range of visual function tests currently used as secondary outcome measures in reported ocular gene therapy trials from **Table 1**.

There was 100% compliance with reporting on the pre-specified primary outcome measures. Sixteen trials pre-specified one or more secondary outcome measure. These were met in full by 68% (11) trials. Five trials did not meet all of the outcome measures but did meet some of them. In addition, 83% (15/18) trials reported additional outcome measures, including a range of features, such as retinal imaging, aspects of visual function, and fMRI imaging. **Figure 1A** demonstrates the number of additional secondary outcomes carried out in the published literature but not originally included in the register as a histogram. **Figure 1B** shows the array of the visual function tests reported. VA was the most commonly used assessment of visual function. Perimetry was also commonly used but could take several different forms; each of which is targeting different areas of the visual field. Mobility testing is not standardized and appears in almost half the published trials. Electroretinograms are measured in a similar number of trials but are standardized due to ISCEV standards. The tests listed in low number of trials are generally non-standardized exploratory techniques such as fixation analysis and dark-adapted perimetry.

## Discussion

Adverse events are a key part of phase 1 trials as would be expected. Visual acuity is also a frequent factor in determining treatment effects and is reported in 94% of trials. It is a widely accepted measure, both clinically and by medical regulatory authorities. VA has been reported to have higher variability in low vision patients so strategies to optimize VA measurements in patients with disease need to be better established (Kiser et al., 2005).

Even within trials for the same disease, visual outcome measures used differ across sites, making direct comparison difficult. This is especially problematic due to the small numbers of patients involved in these highly specialized trials. The ideal way forward would be to conduct formal natural history studies and additional validation studies of novel outcome measures where existing measures are not sufficient or appropriate. The length of natural history studies should be determined by the nature of the disease being investigated. Fast progressing conditions will require a shorter follow-up period of 1 or 2 years. Slower progressive conditions should ideally have a longer follow-up period. An initial audit of imaging and functional data collected in the clinical environment can provide guidance on the speed of progression as a starting point such as conducted by Jolly et al., 2016 and 2017 in choroideremia (Jolly et al., 2016, Jolly et al., 2017). Combining structural and functional data will be helpful in better understanding the disease process as well as treatment impact in both natural history trials as well as in final outcome measures chosen.

Outcome measures should ideally be based on the biology of the disease and related to measurement of the therapeutic target within the eye, in order to maximize the chance of measuring a real therapeutic effect. This may change in end-stage disease *versus* trials designed for early disease states. The balance of structural *versus* functional measures is likely to change in late stage *versus* early stage disease. If the novel outcome measures are established in the disease prior to the interventional trials, the data can be submitted to the regulatory authorities in advance of the interventional trials. This would increase the acceptability of these measures. Outcome measures should be based on an understanding of the underlying disease process as well as the impact of the gene therapy as determined by the expected impact of the viral vector and cells likely to be transfected. A significant advantage of this approach would be the likelihood of reaching the final outcome more quickly due to the use of targeted and sensitive markers of disease.

Patient quality of life is highly dependent on their perception of the world. Subjective assessment using validated instruments can provide insights into visual perception from the patients’ perspective and can be considered as part of the battery of outcome measures (De Boer et al., 2004). Defining success based on clinical (such as repeatability) and patient (such as improvement required for greater quality of life) factors will make a stronger case for the success of therapy, particularly for phase 2 and 3 trials (Mcglothlin and Lewis, 2014). Although questionnaire results may be considered biased due to the patients motivation, the subjective feedback can provide very powerful evidence for the real world impact of any therapy in a way that clinical measures are unable to achieve. Many funding bodies in the United Kingdom encourage the use of patient and participant involvement in research as the insights they can provide can have an influence on guiding researchers to improved clinical trial design, as well as impact when reporting results (Boote et al., 2011).

Despite visual function being highlighted as an important factor in the success of gene therapy trials, little progress has been made on developing a coherent approach worldwide (Thompson et al., 2015). Other fields have highlighted similar issues for gene therapy (Lähteenvuo and Ylä-Herttuala, 2017). As more diseases are targeted by a gene therapy approach, and trials progress to phases 2 and 3, this will become ever more important. Cataract formation is a side effect of the invasive vector delivery techniques (Gupta et al., 2007; Hasler et al., 2015). Moreover, patients are followed up over long periods of time, increasing the chance of age-related cataract formation. Thus, it is necessary to account for the effect of cataract on visual function measures to ensure that any deficits do not interfere with determining the impact of gene therapy. Otherwise, results may be skewed in a negative way masking the therapeutic effect. Greater investment is needed in exploring disease parameters in more detail in order to complete gene therapy trials in an effective, timely, and cost-effective manner. This review provides an important starting point for clinical trial design.

## Author Contributions

JJ and RM contributed conception and design of the study. JJ performed the data analysis and wrote the first draft of the manuscript. HB and RM contributed to manuscript revision, read and approved the submitted version.

## Funding

This study is funded by the National Institute for Health Research (NIHR) [Clinical Doctoral Research Fellowship CA-CDRF-2016-02-002 for Jasleen K Jolly]. The views expressed are those of the authors and not necessarily those of the NHS, the NIHR or the Department of Health and Social Care. The sponsor and funding organization had no role in the design or conduct of this research.

## Conflict of Interest Statement

RM is the scientific founder of Nightstar Therapeutics, receives grant funding from Nightstar Therapeutics, and is a consultant to Nightstar Therapeutics and Spark Therapeutics.

The remaining authors declare that the research was conducted in the absence of any commercial or financial relationships that could be construed as a potential conflict of interest.
