# Supplementary material for: Outcome Measures Used in Ocular Gene Therapy Trials: A Scoping Review of Current Practice
Source: Front Pharmacol. 2019 Sep 18;10:1076. doi: 10.3389/fphar.2019.01076 (PMC6759794; doi:10.3389/fphar.2019.01076)
Supplement: Supplementary file 1 [file Presentation_1.pptx]

## Slide 1
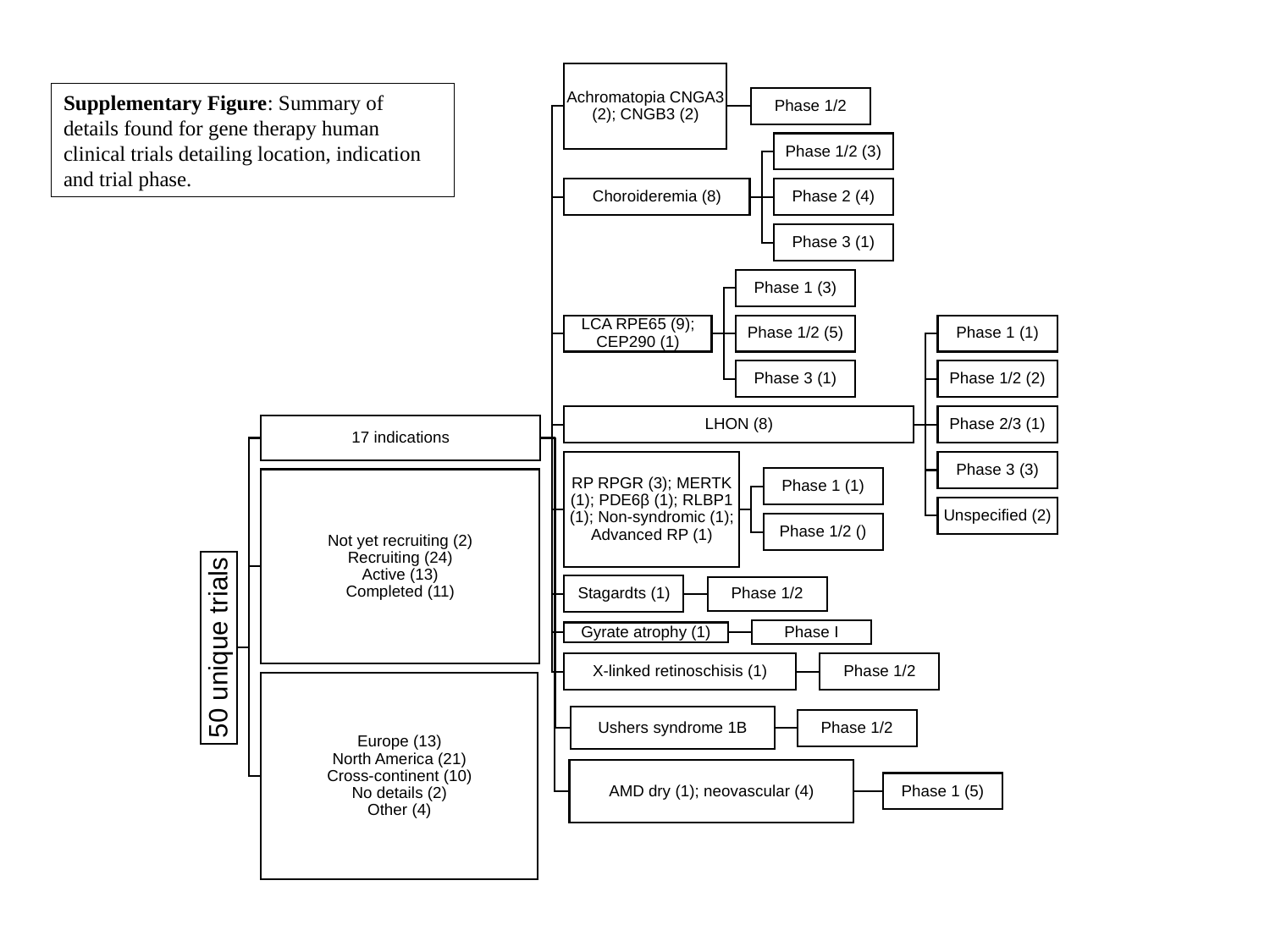

Supplementary Figure: Summary of details found for gene therapy human clinical trials detailing location, indication and trial phase.
