# Supplementary material for: Outcome Measures Used in Ocular Gene Therapy Trials: A Scoping Review of Current Practice
Source: Front Pharmacol. 2019 Sep 18;10:1076. doi: 10.3389/fphar.2019.01076 (PMC6759794; doi:10.3389/fphar.2019.01076)
Supplement: Supplementary file 2 [file Table_1.docx]

**Supplementary Table: Outcome measures on official registered entries for all trials relating to ocular gene therapy.**

| **Phase** | **Primary: Safety measures** | **Primary: Validated tests** | **Primary: Novel tests** | **Primary: Non-specific** | **Secondary: Safety measures** | **Secondary: Validated tests** | **Secondary: Novel tests** | **Secondary: Non-specific** |
| --- | --- | --- | --- | --- | --- | --- | --- | --- |
| I or I/II | Adverse events and laboratory measures, inflammation, maximum tolerated dose, physical health | Visual acuity, IOP, kinetic and static perimetry |  | Photography, tolerability | Conversion disease type, maximum tolerated dose, number of rescue injections | Colour vision, dark adaptation, defined area or features on imaging, ERG, FST, mfERG, questionnaires, reading speed, retinal thickness, perimetry-Goldmann, perimetry-Humphrey, perimetry-microperimetry, perimetry-static, visual acuity distance (logMAR or FrACT), VA near | Dark adaptation cone recovery, light aversion, mobility door task, pupillometry | Efficacy, mobility, objective changes in vision, ophthalmic findings, pupil light reflex, psychophysical tests, quality of life, retinal function, retinal imaging,  visual function, imaging |
| 2 and 3 | Adverse events | Visual acuity | Mobility (binocular) |  | Adverse events, laboratory measures | AF changes, colour vision, contrast sensitivity, FST, OCT ellipsoid zone, OCT RNFL, perimetry-Humphrey, microperimetry, questionnaires, VEP, visual acuity | Mobility (monocular) | AF, computerised visual field, responder analysis |
| Unspecified | Laboratory measures | Visual acuity |  |  | Laboratory measures | ERG, IOP, OCT RNFL, VEP |  | Computerised visual field |
